# *Cinidium officinale* and its Bioactive Compound, Butylidenephthalide, Inhibit Laser-Induced Choroidal Neovascularization in a Rat Model

**DOI:** 10.3390/molecules201119728

**Published:** 2015-11-19

**Authors:** Yun Mi Lee, Yu Ri Lee, Jin Sook Kim, Young Ho Kim, Junghyun Kim

**Affiliations:** 1 College of Pharmacy, Chungnam National University, Daejeon 34134, Korea; candykong@kiom.re.kr (Y.M.L.); yhk@cnu.ac.kr (Y.H.K.); 2 Korean Medicine Convergence Research Division, Korea Institute of Oriental Medicine, Daejeon 34054, Korea; yrsanta@kiom.re.kr (Y.R.L.); jskim@kiom.re.kr (J.S.K.)

**Keywords:** age-related macular degeneration, butylidenephthalide, choroidal neovascularization, *Cinidium officinale*

## Abstract

Choroidal neovascularization (CNV) is a common pathology in age-related macular degeneration. In this study, we evaluated in a rat model the effect of an extract of *Cinidium officinale* Makino and its bioactive compound, butylidenephthalide, on laser-induced CNV. Experimental CNV was induced in Long-Evans rats by laser photocoagulation. *C. officinale* extract (COE) and butylidenephthalide was intraperitoneally injected once per day for ten days after laser photocoagulation. Choroidal flat mounts were prepared to measure CNV areas and macrophage infiltration. We used a protein array to evaluate the expression levels of angiogenic factors. The CNV area and macrophage infiltration in COE-treated rats were significantly lower than in vehicle-treated rats. COE decreased the expression levels of IGFBP-1, MCP-1, PAI-1, and VEGF. Additionally, butylidenephthalide also inhibited the laser-induced CNV formation and macrophage infiltration and down-regulated the expression of IGFBP-1, MCP-1 and VEGF. These results suggest that COE exerts anti-angiogenic effects on laser-induced CNV by inhibiting the expression of IGFBP-1, MCP-1, and VEGF, indicating that anti-angiogenic activities of COE may be in part due to its bioactive compound, butylidenephthalide.

## 1. Introduction

Age-related macular degeneration (AMD) is a leading cause of blindness in the elderly [1]. The majority of patients with AMD have the dry form, which is characterized by the degeneration of retinal pigment epithelial cells and photoreceptor cells. However, more severe vision loss is associated with the wet (neovascular) form [2]. The wet form of AMD is characterized by the growth of blood vessels from the choroid through Bruch’s membrane, resulting in choroidal neovascularization (CNV) in sub-retinal pigment epithelium (RPE) space.

It has been proposed that vascular endothelial growth factor (VEGF) and its receptors play an important role in the progression of AMD [3]. The inhibition of VEGF signaling pathway attenuated the development of CNV in experimental animals [4] and human subjects [5]. Several anti-VEGF agents, such as ranibizumab and bevacizumab, have been shown to markedly suppress neovascular AMD [6]. These VEGF inhibitors have exhibited some efficacy in slowing disease progress and improving vision. However, the intravitreal injection procedure for these drugs occasionally causes several adverse reactions, such as traumatic cataract, endophthalmitis, vitreous hemorrhage, and retinal detachment [7,8]. Therefore, to identify novel agents that inhibit the development of CNV, several drug candidates are under study for possible clinical usage [5,9,10].

*Cinidium officinale* Makino has been used in Asia for centuries as a medicinal plant to treat pain and inflammation. In previous reports, *C. officinale* promoted blood circulation in inflammatory diseases [11,12]. Haranaka *et al.* reported that *C. officinale* has antitumor and antimetastatic activities in experimental animals [13]. Recently, Kwak *et al.* showed that the extract of *C. officinale* inhibited suture-induced corneal neovascularization in rats [14]. In addition, *C. officinale* contains a variety of volatile phthalide derivatives. Butylidenephthalide is one of the major compounds found in *C. officinale* [15]. Butylidenephthalide inhibited human umbilical vein endothelial cell proliferation, migration and capillary-like tube formation *in vitro* and suppressed the development of zebrafish subintestinal vessels *in vivo* [16]. Although anti-angiogenic properties of *C. officinale* and its bioactive ingredient, butylidenephthalide, have been reported, the effect on neovascular AMD is still unknown. Therefore, in this study, we investigated the inhibitory effect of the extract of *C. officinale* and butylidenephthalide on subretinal neovascularization in a rat laser-induced CNV model.

## 2. Results

### 2.1. C. officinale Extract (COE) Inhibits Laser-Induced CNV Formation and Macrophage Infiltration

**Figure 1:**
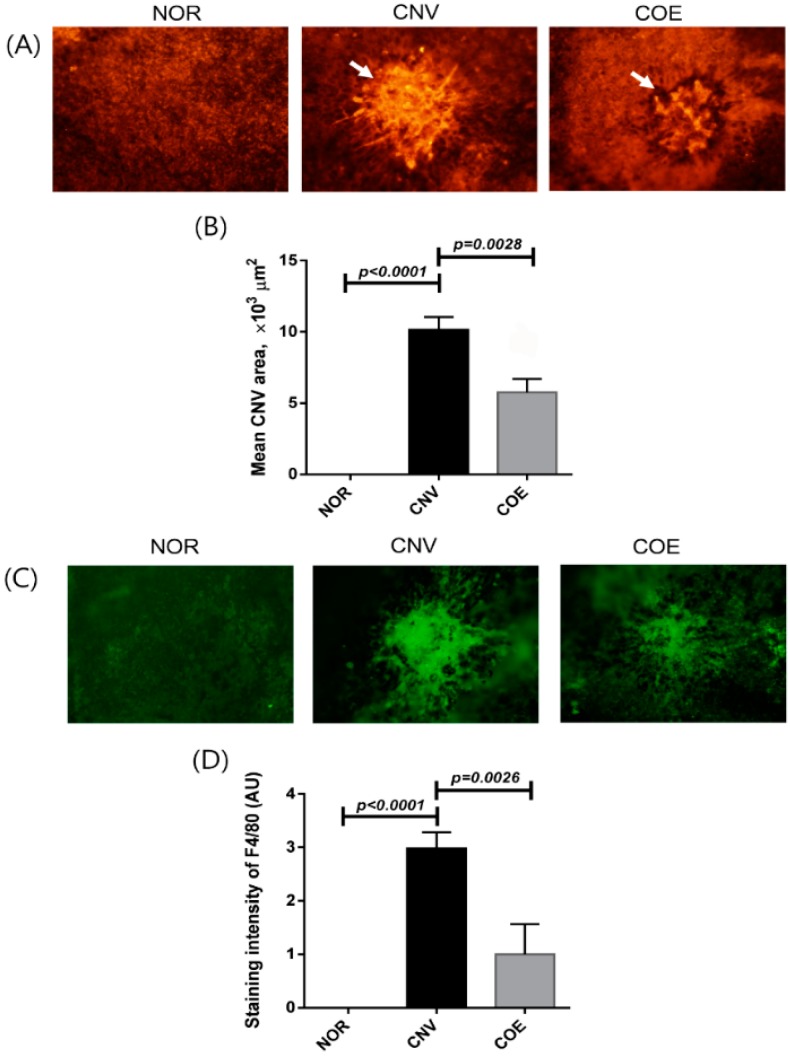
Effect of COE on CNV. (**A**) Choroidal flat mounts of laser-induced CNV. The CNV lesions were labeled with isolectin B4. White arrows indicate laser-induced CNV; (**B**) The areas of CNV lesions were measured in each group; (**C**) The presence of macrophages in CNV was evaluated by immunostaining for F4/80; (**D**) The immunoreactivity of F4/80 in CNV was measured in the choroidal flat mounts. The values in the bar graph represent the mean ± SE, *n* = 5.

The treatment of COE significantly inhibited CNV formation in the subretinal areas. The size of CNV was measured using choroidal flat mounts 10 days after laser photocoagulation. As shown in Figure 1, the mean CNV areas were 11,533 ± 3335 μm^2^ in the vehicle-treated rats and 5845 ± 1730 μm^2^ in the COE 100 mg/kg/day-treated rats.

Rats treated with COE exhibited 49.3% reduction in the extent of CNV lesions compared with the vehicle-treated rats. These results indicate that COE helps to inhibit the laser-induced CNV in rats. To examine how COE suppresses CNV formation, we analyzed the infiltration of macrophages in CNV by immunostaining for the macrophage marker F4/80 (Figure 1C,D). The COE-treated rats showed less immunoreactivity for F4/80 in the RPE-choroid complex compared with vehicle-treated animals. These results suggest that the macrophage infiltration during the process of CNV formation was suppressed by COE.

### 2.2. COE Regulates the Expression of Angiogenesis-Associated Factors

**Figure 2:**
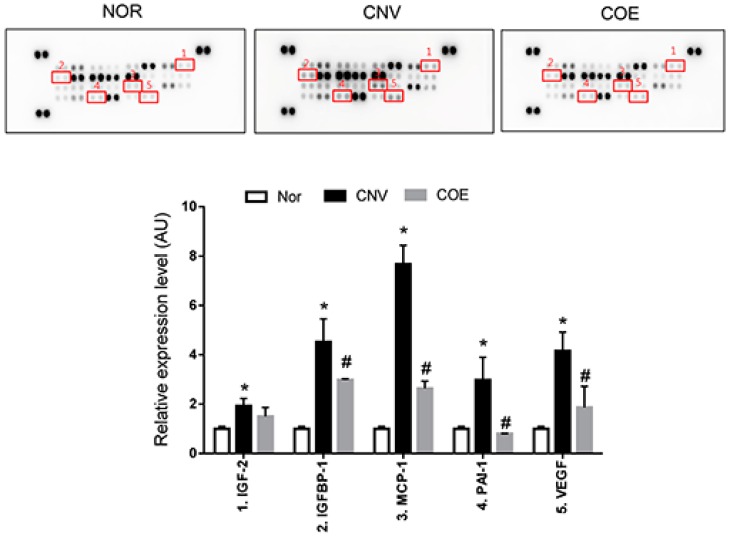
Effects of COE on the expression levels of angiogenesis-related proteins. The positive controls are located in three corners of each array, and the negative control is located in the lower right corner of each array. Modulated proteins in the PRE-choroidal complexes treated with COE are highlighted with squares and indicated by numbers. The values in the bar graph represent the mean ± SE, *n* = 5. * *p* < 0.05 *vs.* normal rats, ^#^ *p* < 0.05 *vs.* vehicle-treated rats.

We investigated the expression levels of angiogenesis-related factors in the RPE-choroidal complexes using a protein array to evaluate the direct effects of COE on CNV. As shown in Figure 2, COE decreased the expression of pro-angiogenic factors (insulin-like growth factor binding protein-1 (IGFBP-1), monocyte chemoattractant protein-1 (MCP-1), plasminogen activator inhibitor 1 (PAI-1) and VEGF) compared with the vehicle-treated rats. The expression of insulin-like growth factor-2 (IGF-2) was significantly increased in the vehicle-treated rats, but this pro-angiogenic factor remained unaffected by COE treatment. The several weak spots in the protein array may be due to low sensitivity of this antibody on the array. These results indicate that COE might exert anti-angiogenic effects by inhibiting the expression of IGFBP-1, MCP-1, PAI-1 and VEGF.

### 2.3. Butylidenephthalide Blocks Laser-Induced CNV Formation

**Figure 3:**
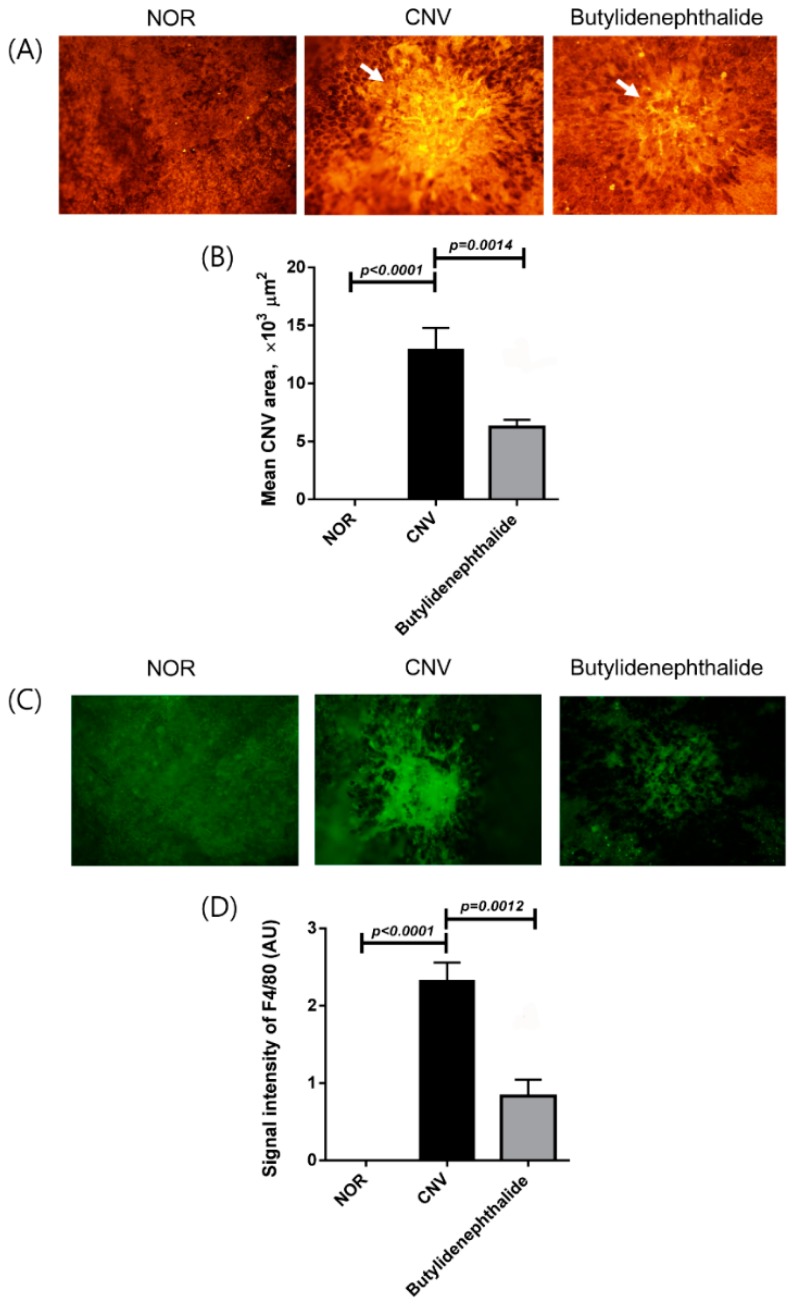
Effect of butylidenephthalide on CNV. (**A**) The CNV lesions were labeled with the endothelial cell marker isolectin B4. White arrows indicate laser-induced CNV; (**B**) The areas of CNV lesions were measured in each group; (**C**) The presence of macrophages in CNV was evaluated by immunostaining for F4/80; (**D**) The immunoreactivity of F4/80 in CNV was measured in the choroidal flat mounts. The values in the bar graph represent the mean ± SE, *n* = 5.

To determine whether butylidenephthalide is a bioactive ingredient of *C. officinale* as an anti-angiogenic agent, this compound was also administered in the rat laser-induced CNV model. As shown in Figure 3, the mean CNV areas were 12,003 ± 4746 μm^2^ in the vehicle-treated rats and 6291 ± 2504 μm^2^ in the butylidenephthalide 5 mg/kg/day-treated rats. Rats treated with butylidenephthalide exhibited 48.6% reductions in the extent of CNV lesions compared with the vehicle-treated rats.

In the immunostaining for the macrophage marker F4/80 (Figure 3C,D), the immunoreactivity for F4/80 tended to be lower in the butylidenephthalide-treated rats than in the vehicle-treated rats.

### 2.4. Butylidenephthalide also Regulates the Expression of Angiogenesis-Associated Factors, Similar to Those Seen in the COE-Treated Rats

**Figure 4:**
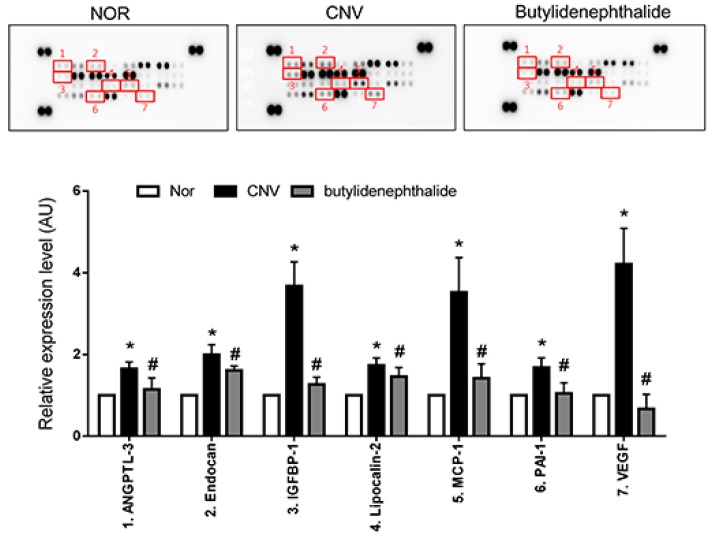
Effects of butylidenephthalide on the expression levels of angiogenesis-related proteins. Modulated proteins in the PRE-choroidal complexes treated with butylidenephthalide are highlighted with squares and indicated by numbers. The values in the bar graph represent the mean ± SE, *n* = 5. * *p* < 0.05 *vs.* normal rats, ^#^ *p* < 0.05 *vs.* vehicle-treated rats.

In the protein array, butylidenephthalide decreased the expression of pro-angiogenic factors (angiopoietin-like 3 (ANGPTL-3), endocan, IGFBP-1, lipocalin-2, MCP-1, PAI-1 and VEGF) compared with the vehicle-treated rats (Figure 4). Among these pro-angiogenic factors, IGFBP-1, MCP-1 and VEGF-1 displayed a >2-fold up-regulation in the vehicle-treated group and a <2-fold down-regulation in the butylidenephthalide-treated group, similar to those seen in the COE-treated rats. These results suggest that butylidenephthalide mediated anti-angiogenic effects by inhibiting the expression of IGFBP-1, MCP-1 and VEGF, indicating that anti-angiogenic activities of COE may be in part due to its bioactive compound, butylidenephthalide.

**Figure 5:**
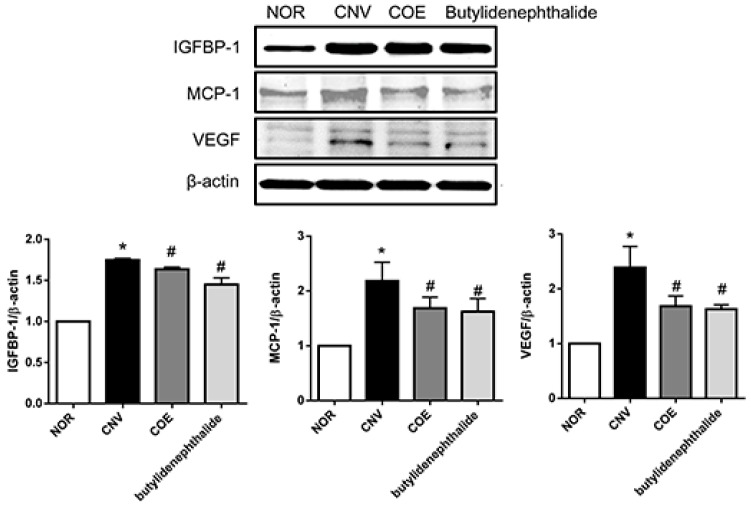
COE and butylidenephthalide suppressed IGFBP-1, MCP-1, and VEGF expression in the RPE-choroidal complex. Protein levels of IGFBP-1, MCP-1, and VEGF were analyzed by Western blotting. The values in the bar graph represent the mean ± SE, *n* = 5. * *p* < 0.05 *vs.* normal rats, ^#^ *p* < 0.05 *vs.* vehicle-treated rats.

To confirm the effect of COE and butylidenephthalide on IGFBP-1, MCP-1 and VEGF in CNV, the expression levels of these proteins were also examined by western blot analysis. Similarly, the protein levels of IGFBP-1, MCP-1, and VEGF were increased compared with the normal control. The decreased levels of these proteins were detected in the COE and butylidenephthalide-treated rats compared with the vehicle-treated rats (Figure 5).

## 3. Discussion

In the present study, we demonstrated for the first time that COE inhibited experimental CNV in a rat model. CNV area and macrophage infiltration were significantly reduced in COE-treated rats compared with vehicle-treated rats. The expression levels of IGFBP-1, MCP-1, PAI-1 and VEGF in the RPE-choroidal complex were lowered by the administration of COE. Furthermore, butylidenephthalide, a bioactive compound found in COE, also significantly suppressed CNV formation and macrophage infiltration and decreased the expression of IGFBP-1, MCP-1, and VEGF-1. In the protein, the expression levels of various angiogenic factors were changed by COE and butylidenephthalide. However, when considering 2-fold change thresholds, IGFBP-1, MCP-1 and VEGF-1 displayed a >2-fold up-regulation in the vehicle-treated rats and a <2-fold down-regulation in the COE and butylidenephthalide-treated group. Taken together, our results suggest that the CNV inhibitory effect of *C. officinale* is primarily via down-regulation of IGFBP-1, MCP-1, and VEGF, and that butylidenephthalide is a potent antiangiogenic bioactive compound of *C. officinale*.

Angiogenesis plays an important role in CNV formation. However, the complex pathogenesis of CNV remains unclear. Normally, the balance between angiogenic and anti-angiogenic factors is tightly controlled [17]. In neovascular AMD, this balance is destroyed, and the overexpressions of pro-angiogenic factors such as VEGF, activate angiogenic pathways and trigger CNV formation [18]. Although the laser-induced CNV rat model is not the same as the CNV secondary to wet AMD in patients, this experimental model shares several same aspects with CNV in patients with wet AMD, including the increased VEGF level, the disruption of Bruch’s membrane and subretinal neovascularization [19]. The murine laser-induced CNV model has been widely used to study neovascular AMD [20]. In this study, we evaluated the therapeutic potential of COE and butylidenephthalide for the treatment of CNV using this animal model.

VEGF inhibitors provide great benefits in patients with neovascular AMD and diabetic retinopathy [21]. However, increasing evidence suggests that MCP-1 and IGFBP-1 also have a role in retinal and choroidal neovascularization. Our protein array indicated that COE and butylidenephthalide markedly suppressed the expression of MCP-1 and IGFBP-1. MCP-1 expression is increased in both wet AMD and diabetic retinopathy. MCP-1 deficiency prevented the development of laser-induced CNV in MCP^−/−^ mice [22]. Moreover, MCP-1 has been shown to contribute to the recruitment of inflammatory cells into the retina [23] and indirectly induces apoptosis in retinal pigment epithelial cells by infiltrating inflammatory cells [24]. Increased expression of IGFBP-1 in the retina also plays an important role in the pathogenesis of retinal neovascularization. Vitreal expression levels of IGFBP-1 were increased in patients with ischemic central retinal vein occlusion [25]. IGFBP-1 shows large increases in neovascular tufts in ischemic retinopathy compared with normal vessels [26]. Based on these findings, we can hypothesize that IGFBP-1 and MCP-1 may be a second validated target against CNV formation [21]. In the present study, COE and butylidenephthalide prevented subretinal neovascularization through down-regulation of IGFBP-1 and MCP-1.

COE has been shown to have multifunctional properties in various experimental models [11,12,13,14]. Onishi *et al.* showed that COE significantly inhibited liver metastasis by colon 26-L5 carcinoma cells and lung metastasis by B16-BL6 melanoma cells *in vivo* [27]. Kwak *et al.* suggested that anti-tumor and anti-metastatic effects of COE might be mediated by its angiogenic activities against neovascularization [14]. Our results suggest that the effects of COE and its bioactive compound, butylidenephthalide, are mediated by IGFBP-1, MCP-1 and VEGF. VEGF acts as a major factor of CNV formation. Other signaling pathways may also be involved in CNV formation. Several previous studies have shown that drugs targeting multiple pathways have more potent anti-angiogenic activities [10]. In this regard, COE and butylidenephthalide are promising agents that may inhibit CNV formation through blocking multiple angiogenic pathways.

Regarding cellular mechanisms for suppressing CNV by the treatment with COE and butylidenephthalide, the present data showed that the treatment led to significant suppression of macrophage infiltration. In previous reports, macrophages, a rich source of VEGF, have been shown to facilitate the development of CNV [28]. It has been reported that *C. officinale* and butylidenephthalide have anti-inflammatory activities [11,12,29]. Collectively, the currently observed suppression of CNV by the treatment with COE and butylidenephthalide is likely attributable to the inhibition of macrophage infiltration and subsequent macrophage-derived VEGF secretion. In conclusion, COE and butylidenephthalide exhibited an anti-angiogenic effect in a rat model of laser-induced CNV. COE and butylidenephthalide suppressed the expression of IGFBP-1, MCP-1 and VEGF. Therefore, COE may serve as a valuable agent to treat human neovascular AMD.

## 4. Experimental Section

### 4.1. Preparation of C. officinale Extract

A standardized COE was purchased from a plant extract bank at that Korea Research Institute of Bioscience & Biotechnology (Daejeon, Korea). A collection of voucher specimens is available for confirmation in that plant extract bank. Briefly, dried and grinded rhizome of *C. officinale* (4.6875 g) was boiled with distilled water for 2 h at 100 °C, and the extract was condensed using freeze-drying (yield: 33.3%). COE was standardized using a reference compound, butylidenephthalide (Sigma, St. Louis, MO, USA), by high-performance liquid chromatography (HPLC, Agilent Technologies, Snata Clara, CA, USA).

### 4.2. Animals and Experimental Design

Seven-week-old male Long-Evans rats were purchased from Japan SLC (Hamamatsu, Japan). Rats were anesthetized using zolazepam (Zoletil, Virbac, Carros, France), and pupils were dilated with 0.5% tropicamide (Santen Pharmaceutical, Osaka, Japan). Experimental CNV was created by laser photocoagulation. Briefly, the fundus was visualized using a microscope cover slip with 0.3% hydroxypropyl methylcellulose (Sigma). A diode laser (Oculight Slx, IRIS Medical, Mountain View, CA, USA) was used for photocoagulation (577 nm wavelength, 0.1 s duration, 100 µm spot size, 150 mW intensity). Four laser spots at equal distance from the optic nerve head were created per eye. Rats that developed cavitation bubbles, indicating rupture of Bruch’s membrane and creation of a sufficient injury to induce CNV, were included in the study. Laser spots with hemorrhagic complications were excluded from further evaluation. The rats in normal control group were maintained without laser photocoagulation. All procedures were approved by the Institutional Animal Care and Use Committee of the Korea Institute of Oriental Medicine (Daejeon, Korea).

### 4.3. Administration of COE and Butylidenephthalide

The laser-treated rats were randomly assigned to three groups: vehicle-only (CNV), COE (100 mg/kg/day) and butylidenephthalide (5 mg/kg/day). The COE and butylidenephthalide was dissolved in 5% DMSO immediately before use, and 100 µL of this solution was injected intraperitoneally once per day for 10 days after laser photocoagulation. The normal control rats (NOR) were injected with the vehicle solution for 10 days. After the treatment with COE and butylidenephthalide, no evidence of systemic adverse effects was observed in any study group.

### 4.4. Preparation of Choroidal Flat Mounts and Lectin Staining

Ten days after laser photocoagulation, rats in each group were anesthetized with zolazepam (Virbac), and eyes were enucleated and fixed in 4% paraformaldehyde for 1 h. The anterior segment was removed and the entire retina was carefully dissected from the eye cup. RPE-choroidal complex was flattened by making four radial incisions with the sclera facing down. The size of CNV lesions was measured in RPE-choroid flat mounts labeled with tetramethylrhodamine isothiocyanate (TRITC)-conjugated isolectin B4 (Sigma). Briefly, the flat mounts were incubated with PBS containing 5% Triton X-100 and 1% bovine serum albumin for 3 h at 37 °C. The flat mounts were then washed 3 times with PBS and labeled with TRITC-conjugated isolectin B4 from *Bandeiraea simplicifolia* (1:50) diluted in PBS. The CNV was viewed with a BX51 microscope (Olympus, Tokyo, Japan). The Image J software (NIH, Bethesda, MD, USA) was used to measure the CNV area.

### 4.5. Angiogenesis-Related Protein Array

To investigate the expression levels of angiogenesis-related proteins in the RPE-choroidal complex, an antibody array analysis (Proteome Profiler™ Rat Adipokine Antibody Array Kit, R & D Systems, Minneapolis, MN, USA) was performed according to the manufacturer’s instructions. Ten days after laser photocoagulation, rats were anesthetized and sacrificed. Each RPE-choroidal complex was carefully isolated under a microscope. The RPE-choroidal complexes were homogenized in PBS using protease inhibitors and centrifuged at 10,000× *g* for 5 min, and the total protein concentrations were quantified. The lysates were added to a membrane spotted with antibodies against angiogenesis-related proteins. After being incubated overnight at 4 °C, the membranes were treated with streptavidin-horseradish peroxidase and visualized using an enhanced chemiluminescence detection system (Amersham Bioscience, Piscataway, NJ, USA) on image analyzer (LAS-3000, Fujifilm, Tokyo, Japan). Optical density measurements were obtained using the Image J software. A list of the antibodies can be found on the manufacturer’s web page [30].

### 4.6. Immunostaining for Infiltrating Macrophages

Ten days after laser photocoagulation, rats were anesthetized and sacrificed. Eyes were enucleated and fixed in 4% paraformaldehyde for 1 h. The anterior segment was removed and the entire retina was carefully dissected from the eye cup. Each RPE-choroidal complex was carefully isolated under a microscope. Whole-mount RPE-choroid complex was incubated with a rabbit polyclonal antibody against macrophage marker F4/80 (Santa Cruz Biotechnology, Paso Robles, CA, USA). The whole mounts were washed for 30 min at room temperature and then incubated for 2 h at 4 °C with fluorescein isothiocyanate-conjugated donkey anti-rabbit immunoglobulin G (Santa Cruz Biotechnology). The RPE-choroid complex was viewed with an Olympus BX51 microscope. Image J software was used to measure the immunoreactivity for F4/80.

### 4.7. Western Blot Analysis

Ten days after laser photocoagulation, rats were anesthetized and sacrificed. Each RPE-choroidal complex was carefully isolated under a microscope. The RPE-choroidal complexes were homogenized in PBS using protease inhibitors and centrifuged at 10,000× *g* for 5 min, and the total protein concentrations were determined by Bradford assay. Exactly equal amounts of protein (50 µg/lane) were loaded, separated by 10% sodium dodecyl sulfate–polyacrylamide gel electrophoresis, and transferred to polyvinylidene difluoride membranes (Bio-Rad, Hercules, CA, USA). The membranes were labeled with mouse anti-IGFBP-1 (Santa Cruz Biotechnology), mouse anti-MCP-1 antibody (Santa Cruz Biotechnology) and mouse anti-VEGF antibody (Abcam, Cambridge, MA, USA). The immunoreactive bands were detected using chemiluminescence detection reagents (Pierce, Rockford, IL, USA), and the density of the bands-of-interest was further measured using a LAS-3000 machine (Fujifilm).

### 4.8. Statistical Analysis

The data are expressed as the mean ± SE. Statistical significance was determined by one-way analysis of variance (ANOVA) followed by Tukey’s multiple comparison test. Differences with *p* < 0.05 were considered statistically significant.
